# Retraction: miR-34 inhibits growth and promotes apoptosis of osteosarcoma in nude mice through targetly regulating TGIF2 expression

**DOI:** 10.1042/BSR-2018-0078_RET

**Published:** 2024-02-28

**Keywords:** apoptosis, miR-34, osteosarcoma, TGIF2, tumor volume

This article is being retracted from *Bioscience Reports* at the request of the authors following receipt of a notification from a reader, alerting the authors and Editorial Office to duplicated images in Figure 2D which have also been published in Feng et al. 2020 (doi: 10.3389/fnana.2020.00036) and similarities in the flow-cytometry data in Figure 4D with multiple unrelated articles.

The authors wish to retract the article. The Editor-in-Chief and Editorial Board agree with the retraction.

